# Direct observation of morphological transition for an adsorbed single polymer chain

**DOI:** 10.1038/s41598-020-77761-0

**Published:** 2020-12-01

**Authors:** Yukari Oda, Daisuke Kawaguchi, Yuma Morimitsu, Satoru Yamamoto, Keiji Tanaka

**Affiliations:** 1 Department of Applied Chemistry, Kyushu University, Fukuoka, 819-0395 Japan; 2 Center for Polymer Interface and Molecular Adhesion Science, Kyushu University, Fukuoka, 819-0395 Japan; 3 International Institute for Carbon-Neutral Energy Research (WPI-I2CNER), Kyushu University, Fukuoka, 819-0395 Japan

**Keywords:** Polymers, Polymer characterization

## Abstract

A better understanding of the structure of polymers at solid interfaces is crucial for designing various polymer nano-composite materials from structural materials to nanomaterials for use in industry. To this end, the first step is to obtain information on how synthetic polymer chains adsorb onto a solid surface. We closely followed the trajectory of a single polymer chain on the surface as a function of temperature using atomic force microscopy. Combining the results with a full-atomistic molecular dynamics simulation revealed that the chain became more rigid on the way to reaching a pseudo-equilibrium state, accompanied by a change in its local conformation from mainly loops to trains. This information will be useful for regulating the physical properties of polymers at the interface.

## Introduction

Polymers have been used in contact with dissimilar solids in a wide variety of industrial applications from traditional uses such as coating agents, adhesives and composites to innovative thin film devices, etc.^1–5^. The performance of the materials and devices is strongly related to the aggregation states and dynamics of polymer chains adsorbed on the solid surface^6–10^, which are essentially different from those in the bulk state^11–20^. It is therefore extremely desirable in the first instance to obtain a better understanding of the fundamental behavior of a single polymer chain as an elemental component to construct the interface with the solid.

The adsorption of polymer chains onto a solid surface from a solution state has been extensively studied both from theoretical and experimental approaches^21–25^, and explained basically in terms of a “loop-train-tail” model^26,27^, where loops and trains are polymer segments having no or direct contact with the surface, respectively, and tails are chain ends with no surface contacts. This model is exquisitely balanced between the attractive interaction between segments and the surface^27,28^ and the conformational and translational entropy loss arisen from the immobilization of a chain with limited conformational freedom onto the surface. The adsorption of chains in the bulk polymer into the solid surface is also an area of extensive research. A general consensus has thus far emerged that there exists a nanometer-thick layer of adsorbed chains at the interface, which cannot be leached out even with a good solvent^29–31^. Great efforts have revealed that adsorbed chains can be categorized into two types, namely strongly- and loosely-adsorbed^32,33^. The former and latter correspond to chains having many trains and loops, respectively. Both types of adsorbed chains are gradually developed to reach a quasi-equilibrium state by thermal annealing, which is accompanied by an elevation of the glass transition temperature of the chains in the adsorbed layer^31,33^. During this process, the fraction of trains in strongly-adsorbed chains increases to overcome the conformational entropic penalty^34^. Thus, once polymer chains are adsorbed onto the solid surface from the solution, it is hypothesized that the chain conformation is transformed from loops to trains, resulting in a more flattened shape, assisted by thermal annealing. Although the experimental collection of evidence at a molecular level is the first step towards finding a method of strengthening the interface, there is currently a lack of such information.

We here tracked the trajectory of a single polymer chain adsorbed on a solid surface, which was trapped in a non-equilibrium state, as a function of temperature using atomic force microscopy (AFM). Although AFM is a powerful tool for ascertaining the dimensions of a single chain at the solid surface^35–37^, discussion about the local conformation at the segment level seems to be limited. To overcome this difficulty, we conducted a full-atomistic molecular dynamics (MD) simulation to interpret the AFM results. As a polymer and solid, a well-defined, or monodispersed, synthetic polymer of methyl methacrylate (PMMA) and a mica with a hydrophilic and molecularly smooth surface were chosen.

## Results

**Figure 1:**
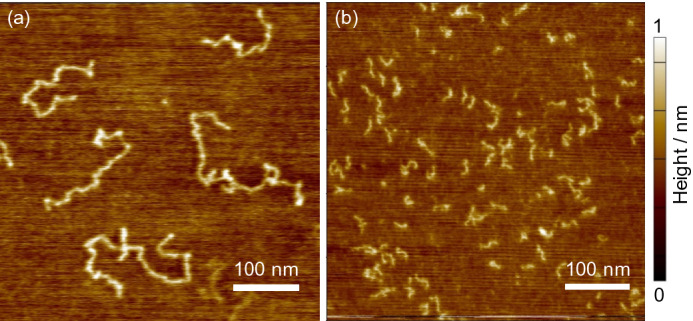
AFM height images for (**a**) PMMA300k and (**b**) PMMA28k chains on mica. Each sample was prepared by spin-coating from 5 × 10^–4^ mg mL^-1^ chloroform solutions and then drying under vacuum at room temperature. Observations were conducted at 298 K in air.

Figure 1a,b show typical AFM height images for PMMA chains with a number-average molecular weight (*M*_n_) of 300 k (PMMA300k) and 28 k (PMMA28k), spin-coated from diluted chloroform solutions onto a mica substrate observed at 298 K. The contour lengths (*L*_exp_) of PMMA300k and PMMA28k chains were 280 ± 50 and 30 ± 2 nm, respectively. They were significantly smaller than the corresponding fully-stretched chain lengths (*L*_n_) of 749 and 70 nm calculated based on *M*_n_. The ratios of *L*_exp_ to *L*_n_ (*r*_L_) were 0.37 ± 0.15 and 0.41 ± 0.16 for PMMA300k and PMMA28k, respectively, and were independent of *M*_n_ within our experimental accuracy. Since the *L*_exp_ was estimated by tracing the shape of a single chain with the eyes using an imaging processing software, it might contain some errors. However, there is no doubt that *L*_exp_ was much smaller than *L*_n_. A plausible reason for the difference between *L*_exp_ and *L*_n_ is the presence of the loop conformation in chains, as predicted by the mean-field approximation theory^27^. PMMA300k was hereafter used unless otherwise stated because longer chains were preferable for the AFM observation.

**Figure 2:**
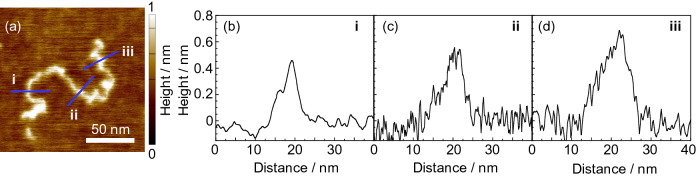
(**a**) An AFM height image for a single PMMA300k chain on mica. Cross-sectional views along lines (**b**) i, (**c**) ii, and (**d**) iii, drawn in (**a**).

Figure 2a shows an AFM height image for a representative single chain on mica. Figure 2b–d show the sectional views along blue lines drawn in panel (a). The maximum height (*h*_max_) for lines i, ii, and iii was 0.46, 0.56, and 0.69 nm, respectively, and the width (*w*) corrected for the tip effect (see Supplementary Information and Fig. S1) was 1.8, 2.5, and 3.5 nm. Since the diameter of PMMA was reported to be 0.7–0.8 nm^38^, the chains seen here look to be slightly flattened and thicker. Also, *h*_max_ and *w* were not always the same along a single chain. Taking into account that the *r*_L_ value was much smaller than unity, as stated in the above, the variation in the height and width, depending on the position, probably reflects the position-dependent local conformation, that is the distribution of trains and loops along the chain.

**Figure 3:**
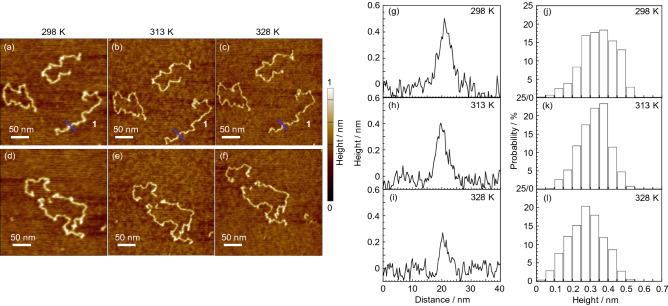
(**a**–**f**) Height images for PMMA300k chains on mica acquired at (**a**,**d**) 298, (**b**,**e**) 313, and (**c**,**f**) 328 K. (**g**–**i**) Cross-sectional views along blue lines for a chain 1 shown in (**a**–**c**). (**j**–**l**) Height histograms for **1** along the contour line at (**j**) 298, (**k**) 313, and (**l**) 328 K.

To examine how a single chain frozen in a non-equilibrium state was adsorbed onto the mica surface during the thermal annealing, chains were observed as a function of temperature. Figure 3a–f show AFM height images for six chains on mica observed at 298, 313, and 328 K. The relative positions for each chain were almost unchanged with increasing temperature, indicating that the centroid of chains was unchanged in this temperature range. On the other hand, the outline for all chains became obviously sharper. Upon closer inspection, the local shape of chains partly changed depending on temperature. We then focused on a single chain, which was marked as **1** in panel (a). Figure 3g–i show the cross-sectional view along the line for **1**. The *h*_max_ and *w* values along the line were 0.50, 0.40, and 0.27 nm and 2.4, 2.0, and 0.7 nm at 298, 313, and 328 K, respectively. Figure 3j–l show the histograms of the height probability for **1** at these temperatures. The height was evaluated by tracing the contour line. The root-mean-square roughness of the substrate remained unchanged at 0.06 nm up to 328 K. The height and width of the chain decreased with increasing temperature, accompanied by an increase in the sharpness of the appearance of the chain. This trend followed for not only **1** but also other five chains (Figs. S2 and S3).

We here considered two effects that are known to affect the visibility of chains at elevated temperatures. First, it has been reported that a condensed water layer exists at the hydrophilic mica surface, and decreases in thickness with increasing temperature^39^. Second, we demonstrated that loops were transformed to trains to increase the contact points between segments and the surface with increasing temperature, as evidenced by the decrease in chain height^40^. Both of these effects were observed here, leading to a suppression of chain mobility and the clear appearance of chains. To address which factor was more dominant, the imaging was again conducted at 298 K after 328 K (Fig. S4). Parts of contour lines, which had become sharper after the heating, became slightly obscured at 298 K. This supports our hypothesis that the evaporation of the condensed water at the surface made the imaging clearer. It is also noteworthy that the imaging of chains at 298 K was much clearer after the heating than before.

The chain mobility generally increases with increasing temperature. This accelerates the transition from mobile loops to immobile trains for chains at the surface. However, we found that even though there was a conformational transition, the chain mobility was somehow suppressed with increasing temperature, which was at variance with the usually observed trend. To confirm this, the AFM observation was conducted at higher temperatures (Fig. S5). The whole shape of chains was almost unchanged even at 393 K compared to that at 298 K except for the imaging clarity, indicating that the chain mobility was still restricted at 393 K. Therefore, it can be claimed that the conformation transition proceeded with increasing temperature.

**Figure 4:**
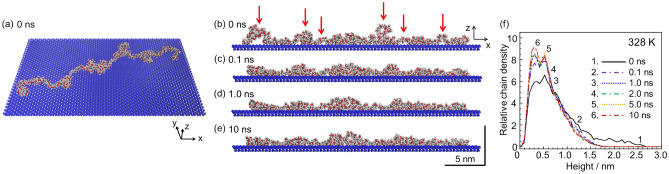
Snapshots of a PMMA28k chain at a hydrophilic surface at 328 K after (**a**,**b**) 0, (**c**) 0.1, (**d**) 1.0, and (**e**) 10 ns. (**a**) Three-dimensional image and (**b**) its projection to the zx plane of the chain where carbon, hydrogen, and oxygen are colored gray, white, and red, respectively. The substrate is colored blue. (**f**) Depth profiles of relative polymer density from the substrate surface at 328 K.

To gain further insight into the conformational transition, an MD simulation was conducted. A single PMMA28k chain with a given conformation was placed on a substrate, relaxed at 298 K for 1 ns and then annealed at 328 K for 10 ns. Figure 4a–e show snapshots of a single chain upon the annealing process. Here, panels (a) and (b-e) show the three-dimensional image and its projection to the zx plane, respectively. The local conformation partly changed with increasing time. In particular, some segments in loops tended to contact with the surface, as marked by red arrows. Figure 4f shows depth profiles of the relative chain density, which were averaged by three simulation results, as a function of time. The relative density increased at a lower height position with increasing time and reached a maximum at a height of approximately 0.3 nm after 10 ns. These trends were consistent with the AFM results.

According to the literature, the average length and fraction of train and loop segments increases and decreases, respectively, upon the adsorption process of polymers from a solution to a solid surface^41,42^. Postulating that such a conformational change also occurs at the solid interface in a polymer film, the development of strongly adsorbed chains with a relative higher density during the film preparation processes could be understood^43–45^. Also, the reorganization of adsorbed chains with a flat conformation is difficult to achieve^46^ or is otherwise extremely slow^41,47^. Since the driving force of the chain adsorption is an attractive interaction with the surface, the increase of segments in the trains makes it easier to cover the entropic penalty of the chain^48^. Thus, once a chain is adsorbed onto the surface with many trains, the conformation is kinetically trapped^49,50^.

The reversibility of the chain adsorption onto the substrate surface is still controversial and, of course, should be dependent on the time/spatial scale as well as temperature^25,51,52^. Although it seems accepted that the adsorption of chains is practically irreversible because the desorption rate is so slow, when the thermal energy associated with rising temperature much increases, adsorbed segments could start to decrease in number^53^. Under experimental conditions in the current study, the adsorption of chains onto the substrate surface could be regarded as an irreversible process. However, the careful reading of Fig. 3 revealed that the shape of the contour lines slightly changed with increasing temperature. This means that while a chain itself could be irreversibly adsorbed, segments could be desorbed and adsorbed and vice-versa. The desorption of a chain from the substrate surface should be energetically studied in the near future^54^.

In conclusion, we studied how a single polymer chain was adsorbed onto a solid surface using AFM together with a full-atomistic MD simulation. The trajectory of a single chain essentially remained unchanged with increasing temperature but the height and width definitely decreased. Concurrently, the imaging of isolated chains became clearer thanks to their restricted molecular motion. The MD simulation strongly supports the molecular picture proposed by AFM that the conformational transition of chains, from loops to trains, proceeded to increase the contact points with the solid surface upon the adsorption process. This represents a key advance in our understanding of the dynamics and interfacial structure of polymers in composite materials including polymer films.

## Methods

As a sample, *syndiotactic*-rich PMMA with *M*_n_ of 300 k and a polydispersity index (PDI) of 1.05 (PMMA300k; Polymer Source Inc.) was used. For comparison, *syndiotactic*-rich PMMA with *M*_n_ of 28 k and PDI of 1.08 (PMMA28k; Polymer Source Inc.) was also used. PMMA was dissolved into chloroform with a concentration of 5 × 10^–4^ mg mL^-1^, which was well below the coil overlapping concentration (c*). Here, c*s for PMMA300k and PMMA28k were calculated to be 34 and 112 mg  mL^-1^, respectively, based on the following Eq. (1);

1 $$c{\text{*}} = \left( {M_{{\text{n}}} /N_{{\text{A}}} } \right)/\left\{\left( {4\pi /3} \right)\cdot R_{\text{g}} ^{{3}}\right\}$$

where *N*_A_ was the Avogadro’s constant. The radius of gyration (*R*_g_) of an unperturbed chain was calculated to be 15.4 and 4.7 nm, for PMMA300k and PMMA28k, respectively, based on the following Eq. (2);

2 $$R_{{\text{g}}} = \left( {N \cdot b^{{2}} /{6}} \right)^{{{1}/{2}}}$$

where *N* and *b* were the degree of polymerization and average statistical segment length, respectively. The *b* value for PMMA was 0.69 nm. Specimens to be observed were prepared onto a freshly-cleaved mica surface by spin-coating the chloroform solutions. They were dried under vacuum at room temperature over 1 h and observed by AFM (Cypher, Asylum Research, Oxford Instruments) with an AC mode using a silicon-made cantilever with a resonance frequency of 2.3–2.4 MHz, a spring constant of 250–280 N m^−1^, and a tip radius of 7 nm in air at 298–393 K. Observations were carried out at least 5 min after heating up/cooling down to a given temperature. The contour line for a single chain was acquired by tracing it with the eyes using the Asylum Research SPM software (ver. 15).

A full-atomistic MD simulation was conducted using the software package Materials Studio 2019 (Dassault Systèmes) with the Forcite module and the COMPASS II force fields. A single chain of *syndiotactic*-PMMA28k was used for calculation. The chain length of a fully-extended form in all-trans conformation of the backbone was 69.7 nm. Starting from the fully-extended form, the structural relaxation was attained under vacuum at 298 K for 100 ps. A random coil conformation was eventually obtained. During the relaxation process, PMMA chains with three different conformations were chosen so that the ratio of chain length to the extended form was comparable to that observed in the experiment. Each chain was placed on a hydrophilic SiO_2_ substrate terminated with OH groups and relaxed at 298 K for 1 ns. Then, the chain was annealed at 328 K for 10 ns to examine the transient change of the adsorbed chain conformation. Depth profiles of the relative polymer density from the substrate surface were obtained by summarizing the existence probability of all atoms composed of PMMA along the direction normal to the surface in 5.5 × 10^–2^ nm steps. The height of each atom was defined as the distance between the center of the atom and the top surface of the substrate along the direction normal to the surface.

## Supplementary information

Supplementary Information.

## Data Availability

All data are available in the main text or the supplementary information.
