# Supplementary material for: Direct observation of morphological transition for an adsorbed single polymer chain
Source: Sci Rep. 2020 Dec 1;10:20914. doi: 10.1038/s41598-020-77761-0 (PMC7708982; doi:10.1038/s41598-020-77761-0)
Supplement: Supplementary file 1 — Supplementary Information. [file 41598_2020_77761_MOESM1_ESM.docx]

Supplementary Information (SI)

**Direct Observation of Morphological Transition for an Adsorbed Single Polymer Chain**

Yukari Oda^†,‡^, Daisuke Kawaguchi^†,‡^, Yuma Morimitsu^†^, Satoru Yamamoto^‡^,

and Keiji Tanaka*^†,‡,§^

*^†^ Department of Applied Chemistry, Kyushu University, Fukuoka 819-0395, Japan*

*^‡^ Centre for Polymer Interface and Molecular Adhesion Science, Kyushu University, Fukuoka 819-0395, Japan*

*^§^ International Institute for Carbon-Neutral Energy Research (WPI-I2CNER), Kyushu University, Fukuoka 819-0395, Japan*

*To whom correspondence should be addressed

FAX: +81-92-802-2880 TEL: +81-92-802-2878

E-mail: k-tanaka@cstf.kyushu-u.ac.jp

**Supporting Data**

**Effect of tip radius on apparent chain width.** The diameter of a polymer chain is apparently broadened in an AFM image by the convolution effect of the tip radius (*R* = 7 nm), a possible value for the real chain radius (*r*) was extracted following the equation (1);^S1^

*D* = 2(*r*•*R*)^1/2^ (1)

where *D* is half of the apparent chain width in the AFM image. Figure S1 shows a schematic illustration of the relationship between a chain radius and a tip radius.


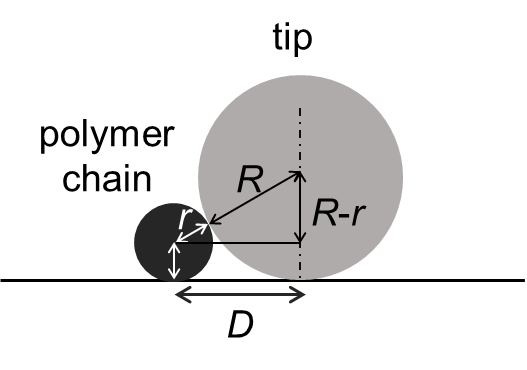


**Figure S1.** A schematic illustration of the relationship between a chain radius and a tip radius.

**Temperature effect on chain conformation.** Figures S2 and S3 show histograms of the height and width populations for PMMA300k chains at 298, 313, and 328 K. The height was estimated by tracing a contour line. The width was estimated by obtaining the cross-sectional view along a contour line at random. Figure S4 shows AFM images for PMMA300k chains acquired at 298 and 328 K, and 298 K after cooling down. Figure S5 shows AFM images for chains acquired at 298, 378, and 393 K.


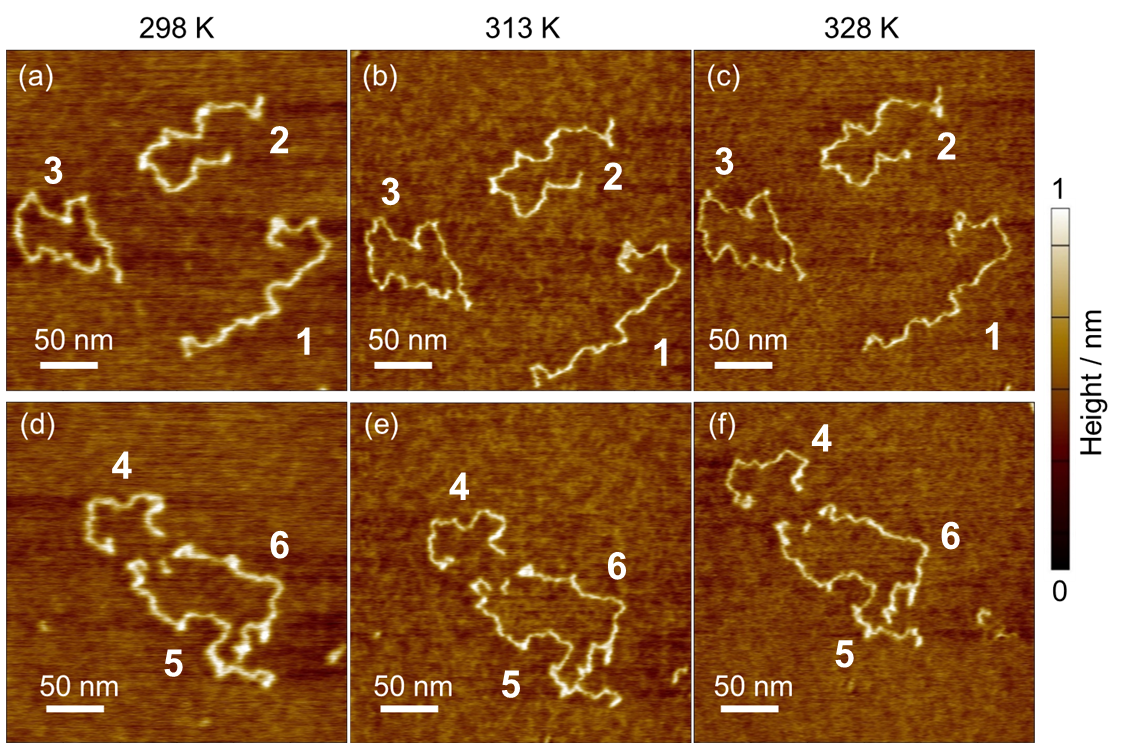


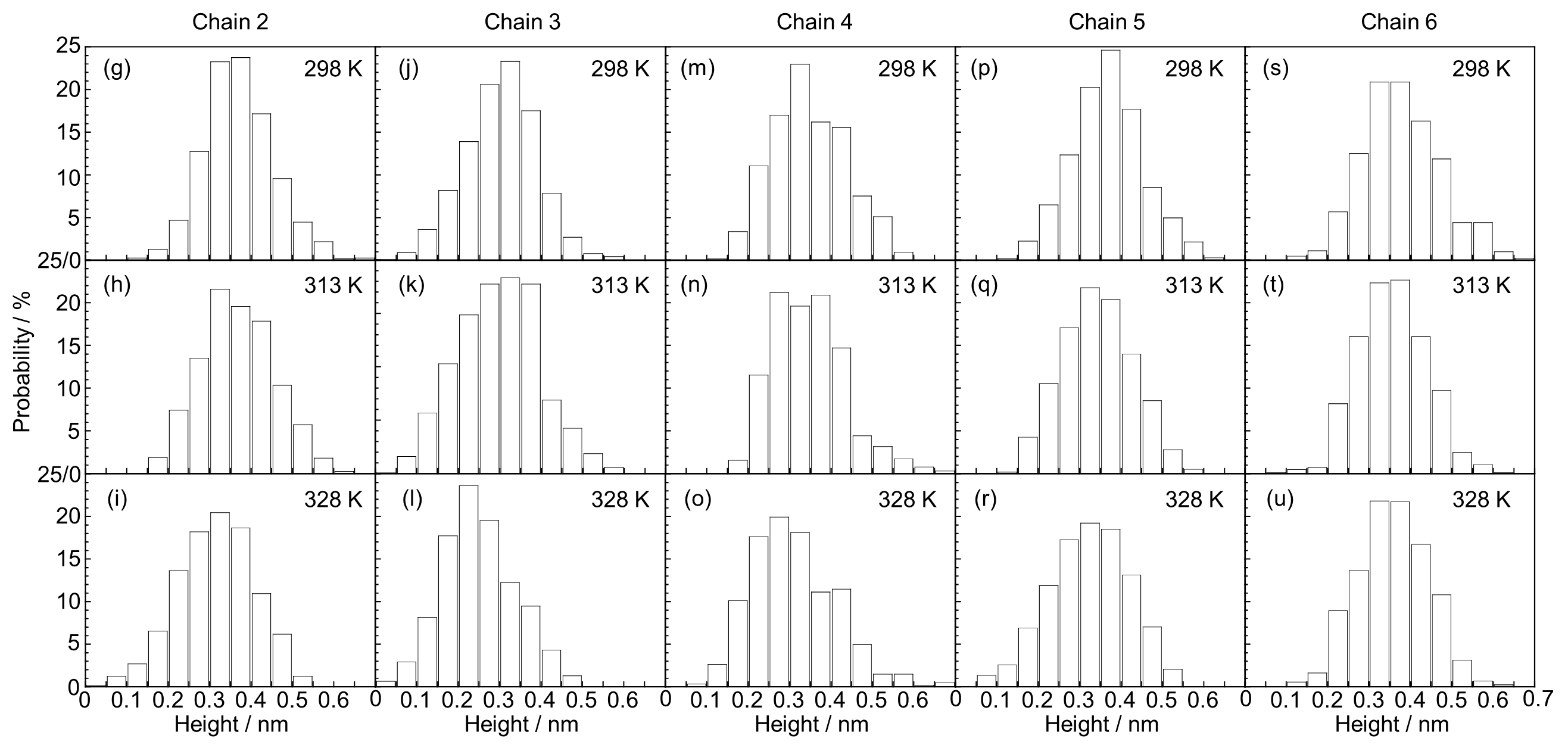


**Figure S2.** (a-f) Height images for PMMA300k chains on mica acquired at 298, 313, and 328 K, which were the same as panels (a-f) of Figure 3. (g-u) Height histograms for chains **2**, **3**, **4**, **5** and **6** shown in panels (a-f) along the contour lines at (g, j, m, p, s) 298, (h, k, n, q, t) 313, and (i, l, o, r, u) 328 K.


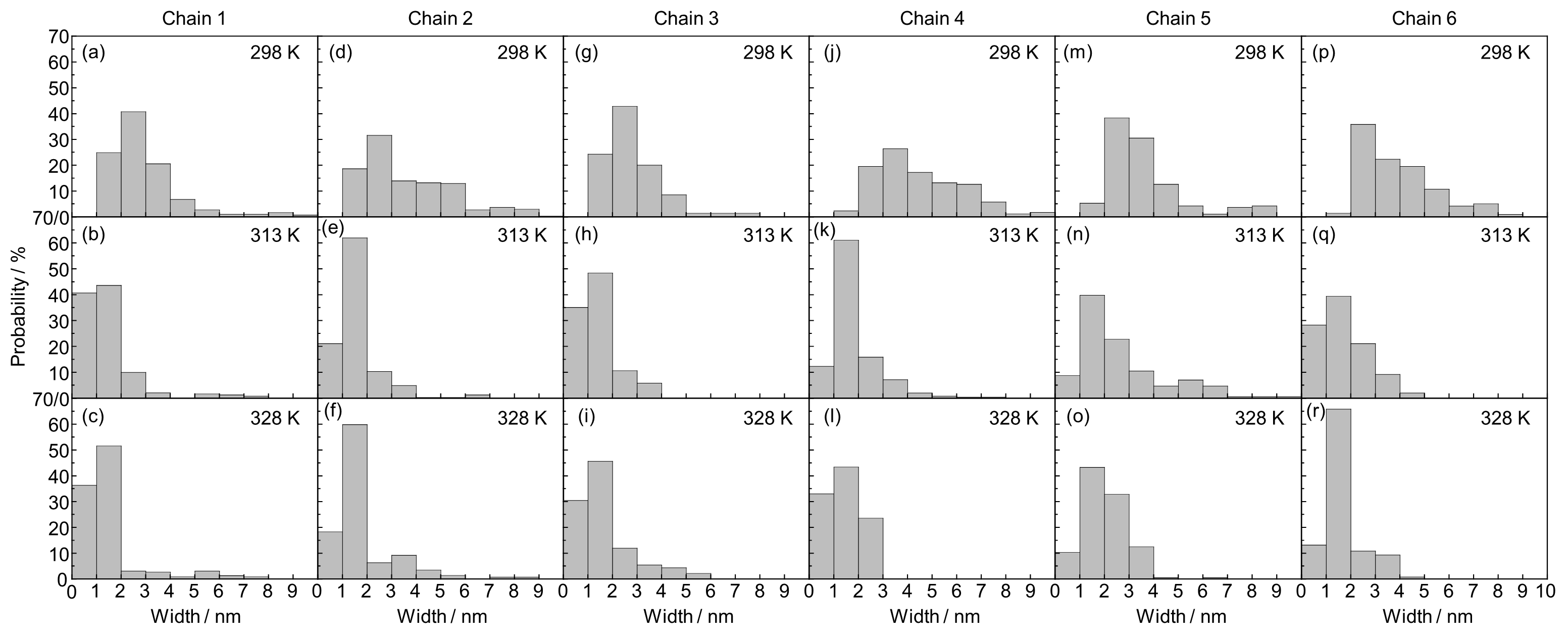


**Figure S3.** (a-r) Width histograms for chains **1**, **2**, **3**, **4**, **5** and **6** shown in panels (a-f) in Figure S2 along the contour lines at (a, d, g, j, m, p) 298, (b, e, h, k, n, q) 313, and (c, f, i, l, o, r) 328 K.


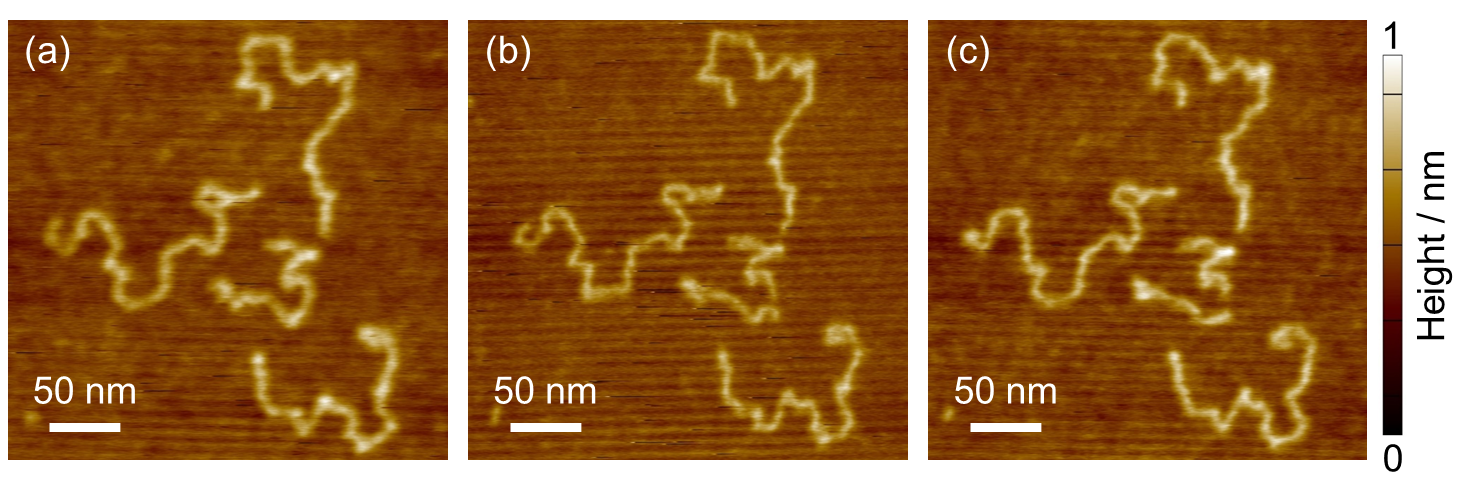


**Figure S4.** AFM images for PMMA300k chains acquired at (a) 298 and (b) 328 K, and (c) 298 K after 328 K.


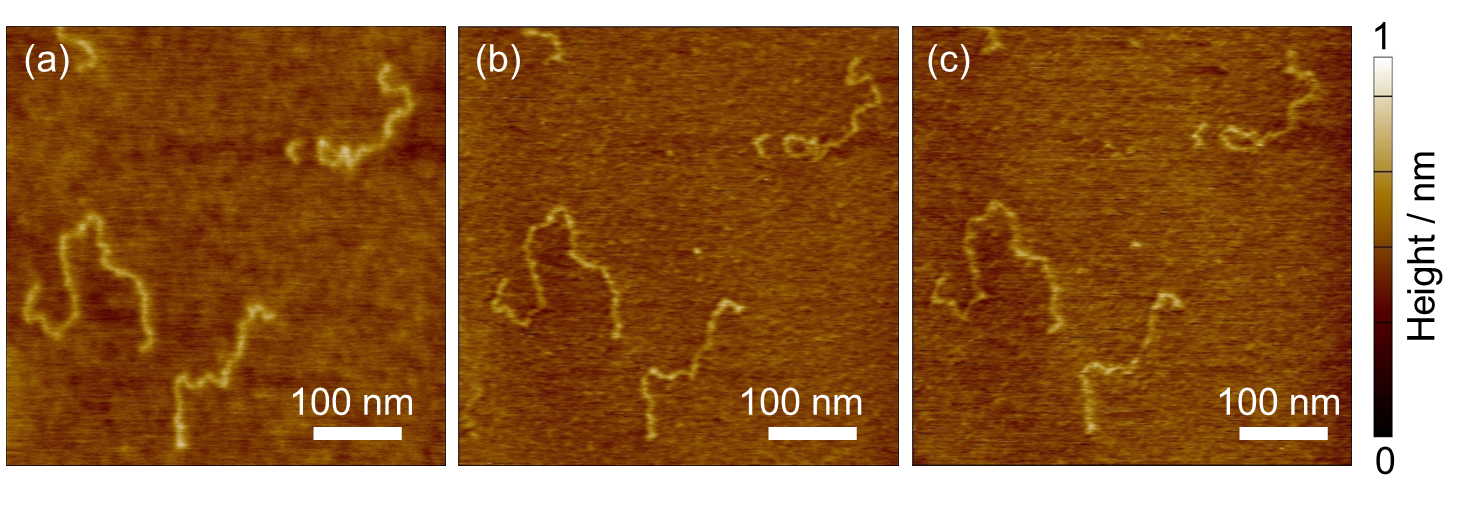


**Figure S5.** AFM images for PMMA300k chains acquired at (a) 298, (b) 378, and (c) 393 K.

**Reference**

S1) Vesenka, J., Guthold, M., Tang, C. L., Keller, D., Delaine, E. & Bustamante, C. Substrate preparation for reliable imaging of DNA molecules with the scanning force microscope. *Ultramicroscopy* **42-44**, 1243-1249 (1992).
